# Lipoxygenase-12 Levels and Biochemical Parameters in Iraqi Patients With Type 2 Diabetes With and Without Benign Prostatic Hyperplasia

**DOI:** 10.7759/cureus.46745

**Published:** 2023-10-09

**Authors:** Bahaa Lateef Kadhim, Khalid Abdulkareem Mohammed

**Affiliations:** 1 Department of Chemistry, College of Science, University of Baghdad, Baghdad, IRQ

**Keywords:** oxidative stress, hyperglycemia, bph, dm2, lipox-12

## Abstract

Background

Diabetes mellitus (DM) is a chronic metabolic disorder characterized by hyperglycemia caused by a defect in the secretion or action or both of insulin. It has a complex pathogenesis. Benign prostatic hyperplasia (BPH) refers to an increase in the size of the prostate; it is one of the most common health problems in men that manifests with age. Lipoxygenase-12 (Lipox-12) is one of the enzymes in the Lipox 12/15 family, which plays a major role in catalyzing a variety of polyunsaturated fatty acids (PUFAs) that are capable of producing different metabolites. Lipox-12 has a significant effect on arachidonic acid metabolism, with PUFA, a pro- and anti-inflammatory mediator, as one of the enzyme isoforms. It also plays a major role in modulating inflammation at multiple checkpoints as diabetes progresses. The present study aims to measure Lipox-12 levels in patients with DM type 2 (DM2) and patients with DM2 + BPH.

Methodology

This study was conducted in Musayyib General Hospital, south of Baghdad, where a clinical examination was performed on 50 samples from controls (healthy subjects), 50 patients with DM2, and 50 patients with DM2 + BPH after taking each patient’s history. The examinations performed included fasting blood sugar (FBS), hemoglobin A1c (HbA1c), prostate-specific antigen (PSA), triglycerides (TG), cholesterol (Chol), and Lipox-12.

Results

The results showed that both the DM2 and DM2 + BPH groups had higher FBS, HbA1c, TG, and Chol levels than healthy subjects; in contrast, Lipox-12 levels were the lowest in the DM2 group (sensitivity = 79% and specificity = 81%) but higher in the DM2 + BPH group (sensitivity = 80%; specificity = 82%) compared to the control group.

Conclusions

Lipox-12 had a high sensitivity and specificity in the DM2 and DM2 + BPH groups compared to the control group, and in both cases, it was used to monitor and diagnose DM2 and BPH.

## Introduction

Diabetes mellitus (DM), a disease in which the metabolism of carbohydrates is affected, decreases the body’s ability to generate or accept insulin, as well as its ability to maintain appropriate levels of glucose in the blood [1,2]. DM type 2 (DM2) is considered to result from the body either not producing enough insulin to meet its needs or the cells not responding to insulin at normal levels [3,4]. In diabetes, the blood vessels associated with increased blood sugar may suffer from oxidative stress due to the imbalance between the production of reactive oxygen species (ROS) and the antioxidant defense systems [5,6]. Unlike healthy people, people with DM2 who are not on any medication are inevitably at risk of developing peripheral vascular disease, stroke, and cardiovascular disease [7].

Benign prostatic hyperplasia (BPH) refers to an increase in the size of the prostate; it is one of the most common problems in men that manifests with age. The prostate is a walnut-sized gland located below the bladder and in front of the rectum, which surrounds part of the urethra. Lower urinary tract symptoms (LUTS) leading to bladder obstruction are caused by BPH and occur due to the following two main components: the static component, which is associated with an increase in benign prostatic tissue that suffocates the lumen of the urethra; and the senile component, which is a dynamic component related to an increase in the urethra [8]. Several factors, such as obesity, lifestyle, growth factors, and ethnicity, have a severe impact on the occurrence of BPH [9]. Among the factors that increase the risk of developing an enlarged prostate is age; depending on age, men are susceptible to moderate or severe prostate enlargement [10].

Lipoxygenase-12 (Lipox-12), an enzyme in the Lipox-12/15 family, plays a major role in catalyzing a variety of polyunsaturated fatty acids (PUFAs) that are capable of producing different metabolites, including 12-hydroxyeicosatetraenoic acid (12-HETE), 15-HETE, and lipoxin (LX), in addition to hepoxilin and protectin, maresin, and resolvin. It also has a significant effect on inflammatory diseases [11], as well as a major role in modulating inflammation at many points of influence, such as the incidence of diabetes. Therefore, reducing inflammatory by-products either through Lipox-12 inhibition or by supplying certain fatty acid substrates through nutritional intrusion will have a significant impact on the outcomes of patients with DM type 1 (DM1) or DM2. Furthermore, Lipox-12 activates islet-inducible deregulation of pro-inflammatory cytokines with greater efficacy than ROS [12].

Hemoglobin A1c (HbA1c), also called glycosylated or glucose hemoglobin, is one of the blood tests that detect DM1 and DM2. It is performed to follow up on the body’s ability to regulate glucose levels in the blood. This test shows the average level of glucose in a person’s blood over two to three months by measuring the percentage of hemoglobin proteins in the blood that glucose encapsulates. Red blood cells have hemoglobin proteins on their surface that have the ability to transport oxygen [13].

## Materials and methods

**Figure 1:**
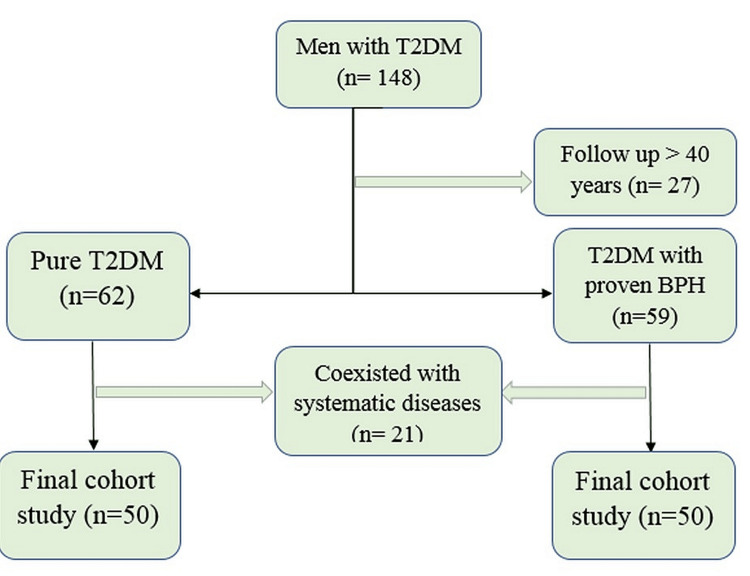
Flowchart illustrating the study design. BPH = benign prostatic hyperplasia; T2DM = type 2 diabetes mellitus

This study was conducted from November 2022 to February 2023 in Musayyib General Hospital, south of Baghdad, with institutional review board number IEC/2022/31. Only participants over the age of 40 years with confirmed DM2 with or without BPH were included in this study. Participants with high blood pressure, malignancies, and contagion (inflammation of the urethra and liver) were excluded from this research. The social and medical history of each patient was obtained as required. The clinical examination was conducted on the following three groups: the first group comprised healthy volunteers who had no past record or signs of DM, BPH, or any chronic diseases such as hypertension. The second group consisted of patients diagnosed with DM, while the third group consisted of patients diagnosed with both DM and BPH. The test was performed on 150 participants aged between 50 and 77 years, which included the control group of 50 participants, the DM2 group of 50 participants, and, finally, the DM2 + BPH group of 50 participants (Figure 1).

The tests conducted on the patients included fasting blood sugar (FBS) measured by Accu-Chek Aviva (Roche, Switzerland), HbA1c measured by DCA Vantage Analyzer (Siemens, Germany), prostate-specific antigen (PSA) measured by IMMULITE 2000 PSA assay kit (Siemens, Germany), triglycerides (TG) measured by Stanbio Triglyceride LiquiColor Test (EKF Diagnostics, UK), cholesterol (Chol) measured by Cholesteryl Ester Quantitation Kit (Sigma-Aldrich, USA), and Lipox-12 measured by ALOX12 ELISA Kit (Elabscience ltd, India). Serum samples were obtained from patients in all groups (DM, DM + BPH, and normal control) to test for these parameters.

The sample size was calculated using the following formula: n = Z^2^⋅σ^2^​/E^2^, where n = required sample size; Z = Z-score corresponding to the desired level of confidence (e.g., 1.96 for a 95% confidence level); σ = estimated population standard deviation; and E = desired margin of error (the maximum acceptable difference between the sample mean and the population mean).

Biochemical assays

The samples (10 mL of the patient’s blood) were taken to the laboratories at Musayyib General Hospital, where it was split into two parts: 2 mL was examined with ethylenediaminetetraacetic acid (EDTA). The levels of serum Lipox-12 were measured using enzyme-linked immunosorbent assay (ELISA) kits according to the manufacturer’s recommendation. For this, 8 mL of venous blood was collected and centrifuged at 3,000 rpm for 15 minutes. The obtained serum was incubated at 25°C for 10 minutes. The serum was split further into two parts: one part was used for the assessment of FBS, Chol, TG, and PSA, while 2-2.5 mL was stowed at 2-8°C for the determination of Lipox-12. Finally, 2 mL of whole blood was collected in an EDTA tube and the level of HbA1C was measured using the Boronate affinity chromatography method.

Hemoglobin A1c

The sample (whole blood) for this test was obtained from the patients by pricking the tip of the finger with a small needle and taking a drop of blood for bedside examination or drawing blood from a vein and sending it to the laboratory for analysis. This test does not require fasting and can be done every three months. It aids in the diagnosis of pre-diabetes (in case of a slight increase in blood sugar) as well as DM1 and DM2 (in case of excessively high blood sugar).

Fasting blood sugar, cholesterol, and triglycerides

Serum was used for these tests. Three tubes labeled “blank,” “sample,” and ”standard” were prepared at room temperature, and a reagent was added to all of them; serum was only added to the tube labeled “sample,” and the standard was only added to the tube labeled “standard.” After mixing, the tubes were left at room temperature for 10 minutes or at 37°C for five minutes. The absorbance of the sample was measured against the blank at 500 nm.

Prostate-specific antigen

The standard working solution process was performed by placing it in the first and second columns, after which the solution was placed in two parts in each of the two wells, one adjacent to the other. The samples were then placed in the other wells and the plate was covered with an airtight lid and incubated for 90 minutes at 37°C.

Next, the liquid was poured from the wells without washing; then, the biotinylate reagent was poured into all the wells and covered with a transparent lid. This was then mixed slightly and incubated for an hour at 37°C. Next, the solution was poured from the wells, and a buffer solution was added to all the wells and left for two minutes. After, the solution was poured and left to dry on absorbent paper and washed three times. Horseradish peroxidase conjugate solution was poured into all the wells, covered with a transparent lid, and incubated for 60 minutes at 37°C. Then, the solution was poured from the wells and washed five times as before. A substrate reagent was added to all the wells, which were then covered with a transparent lid, incubated for 15 minutes at 37°C, and kept on a plate without exposure to light. Finally, the stop solution was added to the wells, and the optical density of the wells was read in one go through a plate reader at 450 nm.

Statistical analysis

The obtained data was statistically analyzed using the SPSS software version 26.0 (IBM Corp., Armonk, NY, USA). The obtained data was presented as means ± SD. One-way analysis of variance was used to make a statistical comparison either within patient groups or with the control group by testing the significance level of differences in means between more than two groups. A p-value of less than 0.05 was considered statistically significant. We used SPSS software to produce only p-values from the entered data to determine the association between the patients and control groups. However, the odds ratio was already calculated by the software to reach the p-value.

## Results

All parameters in this study were compared between the BPH + DM2, DM2, and control groups. Table 1 presents a comparison of HbA1c between all groups.

**Table 1:** Comparison of HbA1c between all groups. BPH = benign prostatic hyperplasia; DM = diabetes mellitus; SD = standard deviation; HbA1C = hemoglobin A1c

Parameter	BPH + DM2 (mean ± SD)	DM2 (mean ± SD)	Control (mean ± SD)	P-value
HbA1c (%)	8.2 ± 2.7	7.9 ± 1.4	5.87 ± 1.2	0.030

The BPH + DM2 group had the highest mean HbA1c value (8.2 ± 2.7), followed by the DM2 group (7.9 ± 1.4); the control group had the lowest mean HbA1c value (5.87 ± 1.2). The p-value of 0.030 indicates that this difference was statistically significant, suggesting that the BPH + DM2 and DM2 groups had significantly higher HbA1c levels than the healthy control group. Because of the direct and positive association between HbA1c and these two diseases, this finding implies that individuals with both BPH and DM2 may have higher HbA1c levels compared to individuals with DM2 alone or individuals without either of these conditions (control group). Therefore, an increase in HbA1c increases the severity and progression of the disease.

Table 2 explains the comparison between all groups according to FBS, PSA, Chol, and TG.

**Table 2:** Comparison between all groups according to FBS, PSA, Chol, and TG. FBS = fasting blood sugar; PSA = prostate-specific antigen; TG = triglycerides; Chol = cholesterol

Parameter	BPH + DM2 (mean ± SD)	DM2 (mean ± SD)	Control (mean ± SD)	P-value
FBS(mg/dL)	132.4 ± 15.7	130.4 ± 10.2	101.5 ± 20.7	0.032
PSA (ng/dL)	5.1 ± 2.41	2.61 ± 1.7	1.99 ± 1.2	0.009
Chol (mg/dL)	218.6 ± 11.2	223.8 ± 10.9	179.2 ± 11.8	0.025
TG (mg/dL)	197.4 ± 20.7	182.4 ± 19.1	134.5 ± 21.1	0.019

There were noticeable changes according to FBS between all groups: BPH + DM2 (mean ± SD) (132.4 ± 15.7), DM2 (mean ± SD) (130.4 ± 10.2), and controls (mean ± SD) (101.5 ± 20.7). There were clear changes according to PSA between all groups: BPH + DM2 (mean ± SD) (5.1 ± 2.41), DM2 (mean ± SD) (2.61 ± 1.7), and controls (mean ± SD) (1.99 ± 1.2). There were slight changes according to Chol between all groups: BPH + DM2 (mean ± SD) (218.6 ± 11.2), DM2 (mean ± SD) (223.8 ± 10.9), and controls (mean ± SD) (179.2 ± 11.8). There were clear changes according to TG between all groups BPH + DM2 (mean ± SD) (197.4 ± 20.7), DM2 (mean ± SD) (182.4±19.1), and controls (mean ± SD) (134.5 ± 21.1).

Table 3 explains the comparison of Lipox-12 levels between all groups.

**Table 3:** Comparison of Lipox-12 levels between all groups. BPH = benign prostatic hyperplasia; DM = diabetes mellitus; SD = standard deviation

Parameter	BPH + DM2 (mean ± SD)	DM2 (mean ± SD)	Control (mean ± SD)	P-value
Lipox-12 (ng/dL)	0.53 ± 0.51	0.45 ± 0.43	0.82 ± 0.15	0.018

Lipox-12 levels were compared among the three groups, and the mean ± SD values for Lipox-12 levels were 0.53 ± 0.51 ng/dL, 0.45 ± 0.43 ng/dL, and 0.82 ± 0.15 ng/dL in the BPH + DM2, DM2, and control groups, respectively. Lipox-12 was associated with inflammatory processes, and the differences in Lipox-12 levels observed between the groups may reflect differences in the inflammatory status of each group.

It is clear from the table that Lipox-12 had a lower effect on the DM2 group compared to the controls. Lipox-12 >0.66 was good for DM2. The cut-off value for Lipox-12 had a sensitivity of 79% and a specificity of 81% with a total area under the curve (AUC) of 0.890, through which subjects can be classified into satisfactory and healthy conditions (Table 4).

**Table 4:** Sensitivity, specificity, and cut-off value for Lipox-12. AUC = total area under the curve

Parameter	AUC	Sensitivity (%)	Specificity (%)	Cut-off value
Lipox-12 (ng/mL)	0.890	79	81	0.66

Lipox-12 is impacted by the BPH + DM2 disease because it binds through the highest receiver operating characteristic curve (ROC) surface area in the framework of differentiation case and control (Table 5).

**Table 5:** Sensitivity, specificity, and cut-off value for Lipox-12. AUC = total area under the curve

Parameter	AUC	Sensitivity (%)	Specificity (%)	Cut-off value
Lipox-12 (ng/mL)	0.788	80	82	0.64

## Discussion

Lipox-12 is a member of the lipoxygenase family responsible for the oxygenation of cellular PUFAs to produce lipid mediators that modulate cell inflammation [11]. The current literature survey for Lipox-12 reveals that the level of this enzyme increases in DM patients and is implicated in the complication of diabetic retinopathy as it has a vascular complication property. To date, there is no published study studying 12-Lipox enzyme levels in patients with DM + BPH disease.

DM is a chronic disease with two different forms depending on whether there is insulin resistance or poor insulin secretion. Additionally, there is evidence showing that chronic inflammation and oxidative stress are involved in DM2 and its complications [14]. Oxidative stress plays a major role in the development of diabetes and its complications, and it is also associated with several pathological conditions, including cancer, diabetes mellitus, neurodegenerative disease, sleep apnea, atherosclerosis, and rheumatoid arthritis. Irregularly high blood sugar leads to neuropathy and retinal disease, as well as complications in the kidneys, liver, and heart. Hyperglycemia stimulates free radicals and impairs the self-defense antioxidant systems through various mechanisms [15].

BPH is the disease that leads to LUTS and is one of the most common problems affecting the urinary tract in males over 50 years old [16]. There are several modifiable risk factors for BPH, including obesity and metabolism. A common condition in older men is BPH with chronic renal failure [17]. The latter is a well-described complication of BPH. The main cause of this disease is not known, but its occurrence depends on several factors, including persistent inflammation, age, lifestyle, and bacterial infection [18].

The role of Lipox-12 in groups of patients in this study

The lipoxygenases catalyze the oxygenation of cellular enzymes that lead to the formation of metabolites of PUFAs that can act in inflammatory pathways such as the eicosanoid, paracrine, autocrine, and endocrine pathways [19]. Lipox acts by stimulating the metabolism of many PUFA substrates (including arachidonic acid, linoleic acid, dihomo-γ-linoleic acid, eicosapentaenoic acid, and docosahexaenoic acid) to form several biologically active products that participate in protective and anti-inflammatory activity. PUFAs play an important role in cellular homeostasis and have also been the subject of research interest for several years [20]. Lipox-12 metabolizes lipids that can be expressed in metabolically active tissues and that regulate cellular processes and their eicosanoid products, including HETE-12. Eicosanoids function to regulate inflammation, cellular turbidity, platelet aggregation, and vascular permeability. This study’s results confirm a decrease in Lipox-12 levels in the patient groups (DM2 + BHP with DM2 groups) compared to the control group. This reduction increases the risk of some fatty acid accumulation due to impaired PUFA metabolism. The accumulation of fatty acids in tissue can cause oxidative stress that leads to disease complications. However, this study disagrees with a previous study [21] that reported an increase in Lipox-12 in patients with DM2 but agrees with another study [22] that demonstrated a reduction in Lipox-12 levels in patients with BPH. Our study concluded that the reduction of Lipox-12 in patients with DM2 and BPH supports fatty accumulation in tissues, causing an increase in oxidative stress products that lead to disease complications [23].

The role of fasting blood sugar and hemoglobin A1c in the groups of patients in this study

The HbA1c test shows the average blood glucose or its control rate in the blood for a period of two to three months. This is determined based on the adhesion of glucose to hemoglobin, which is the protein on red cells that has a lifespan of about three months [24]. Although HbA1c is not considered an accurate measure of chronic hyperglycemia, it is a good predictor of the risk of complications of diabetes in the future [25]. High HbA1c levels can be considered a risk indicator for coronary heart disease and stroke in people with and without diabetes. Therefore, the HbA1c test is accurate for predicting and diagnosing DM. It has also been noted that patients with diabetic retinopathy have a high level of HbA1c. This study’s results confirm an increase in FBS and HbA1c levels in the patient groups (DM2 + BHP and DM2 groups) compared with the control group. This increase allows the accumulation of some fatty acid types due to insulin resistance by cells and increases the glycation process of hemoglobin. Moreover, the accumulation of glucose in the body can generate oxidative stress products that can lead to disease complications and support cell proliferation in the prostate gland. This study agrees with a previous study [26] that confirmed that an increase in glucose levels can lead to the development of prostatic hyperplasia in patients with DM.

Sensitivity and specificity of Lipox-12

By conducting a statistical analysis according to the sensitivity and specificity of Lipox-12 and comparing this metric between the groups, the following findings were obtained [27].

A high AUC was observed when comparing the sensitivity and specificity of Lipox-12 between the control group and the DM groups. It also turns out that Lipox-12 is a new marker that has high sensitivity and specificity when compared, and it can be used as a marker for monitoring patients with DM as well as diagnosing DM [28].

Conversely, when comparing the DM group to the BPH group, the study revealed notably elevated values for sensitivity, specificity, and the AUC for Lipox-12. These findings suggest that Lipox-12 may also serve as a valuable tool for monitoring and diagnosing prostate enlargement (BPH).

This study has some limitations. This study did not involve young or adult people and had a small sample size. A larger sample size would have provided more reliable results. Multi-center studies involving diverse populations would help validate our results. The study did not account for potential confounding factors that could influence Lipox-12 levels, such as age, body mass index, comorbidities, medication use, and lifestyle factors. These factors may have an impact on the results and should be considered in future studies. The absence of an oral glucose tolerance test to diagnose DM2. The study focuses on the measurement of Lipox-12 levels but does not provide detailed mechanistic insights into its role in the pathogenesis of DM2 and BPH. Conducting larger studies with different populations, considering various influencing factors, and exploring the mechanisms behind Lipox-12 could enhance the findings. Future studies are needed to explore the relationship between Lipox-12 and its metabolite 12-HETE in diabetic retinopathy patients suffering from BPH. Furthermore, future studies should also focus on the mechanism and effect of 12-Lipox and 12-HETE in BPH patients with diabetic retinopathy.

## Conclusions

According to the results, patients with only DM or both DM and BPH showed decreased levels of 12-Lipox compared to healthy individuals. Therefore, patients with DM + BPH are more exposed to arteriosclerosis.
